# Supplementary material for: Ligand Identification using Deep Learning
Source: bioRxiv. 2024 Aug 28:2024.08.27.610022. Preprint. [Version 1] doi: 10.1101/2024.08.27.610022 (PMC11383698; doi:10.1101/2024.08.27.610022)
Supplement: Supplementary file 1 [file NIHPP2024.08.27.610022v1-supplement-1.pdf]

bioRxiv preprint doi: https://doi.org/10.1101/2024.08.27.610022; this version posted August 28, 2024. The copyright holder for this preprint (which was not certified by peer review) is the author/funder, who has granted bioRxiv a license to display the preprint in perpetuity. It is made available under aCC-BY 4.0 International license.

Ligand Identification using Deep Learning: Supplement

Jacek Karolczak[1,†], Anna Przybyłowska[1,†], Konrad Szewczyk[1,†], Witold Taisner[1,†], John M. Heumann[2], Michael H.B. Stowell[2], Michał Nowicki[3] and Dariusz Brzezinski[1,*]

[1]Institute of Computing Science, Poznan University of Technology, Piotrowo 2, 60-965 Poznan, Poland, [2]Department of Molecular, Cellular and Developmental Biology, University of Colorado Boulder, Boulder, CO 80309, USA, [3]Institute of Robotics and Machine Intelligence, Poznan University of Technology, Piotrowo 3A, 60-965 Poznan, Poland.

†Equal contribution.

*To whom correspondence should be addressed.

List of supplementary figures:

- **Fig S1**. Predictive performance of CheckMyBlob for X-ray crystallography ligands.
- **Fig S2**. Classification accuracy and number of predictions of MinkLoc3Dv2 and CheckMyBlob within different prediction probability groups.

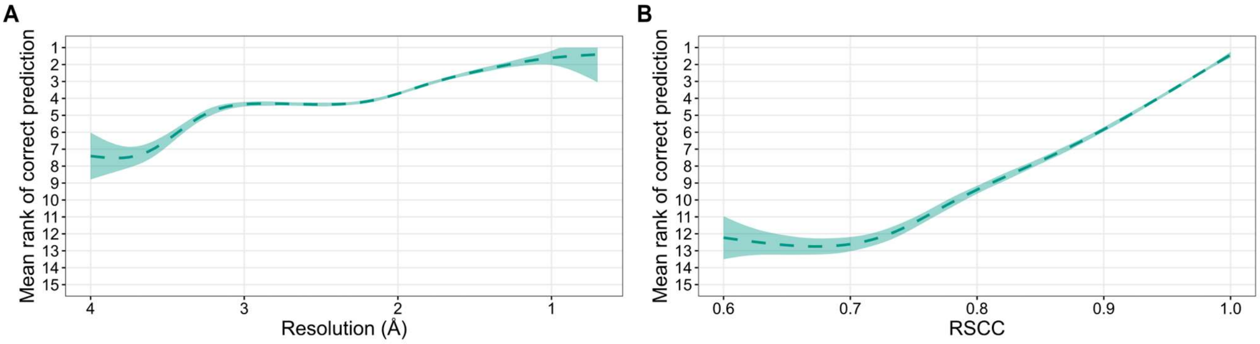

Fig. S5. Predictive performance of CheckMyBlob for X-ray crystallography ligands. Each plot shows the mean ranks of the correct ligand (lines with 95% confidence interval bands) versus (**A**) resolution and (**B**) real-space correlation coefficient (RSCC). The trends for both plots are almost identical to those observed for the MinkLoc3Dv2 model (Fig. 3 in the main text).

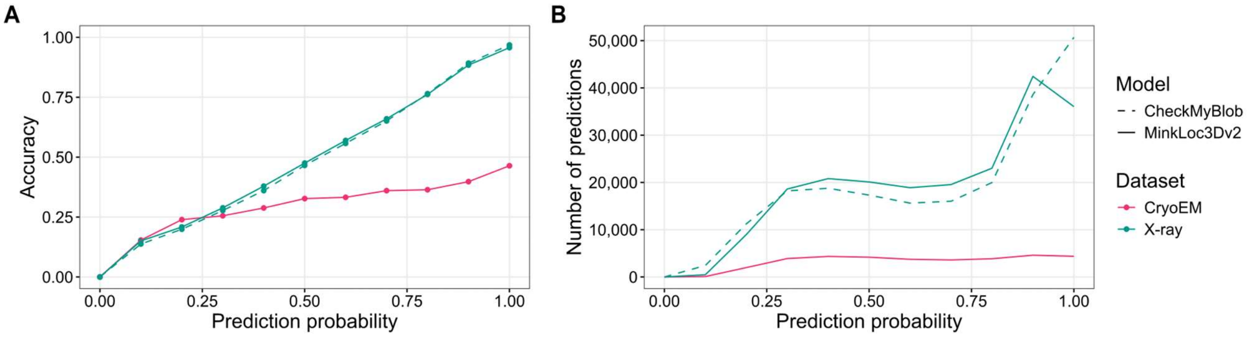

Fig. S6. Classification accuracy and number of predictions of MinkLoc3Dv2 (solid line) and CheckMyBlob (dashed line) within different prediction probability groups. (**A**) The models are well-calibrated on the X-ray diffraction data (green), whereas the higher prediction probabilities for cryoEM (pink) are overestimated. (**B**) The numbers of predictions for X-ray data show a clear distinction between unsure ligand (plateau around 0.5 probability) and confident predictions (spike around 0.9 probability). The cryoEM predictions do not show this distinction.
